# HPV‐Related Cancers in Solid Organ Transplant Recipients: A Nationwide Danish Cohort Study

**DOI:** 10.1002/ijc.70529

**Published:** 2026-04-28

**Authors:** Signe Timm, Flemming Skjøth, Torben Frøstrup Hansen, Lars Henrik Jensen, Paw Christian Holdgaard, Lars Ulrik Fokdal, Torben Bjerregaard Larsen, Mette Moeller Soerensen

**Affiliations:** ^1^ Department of Oncology Vejle Hospital, University Hospital of Southern Vejle Denmark; ^2^ Institute of Regional Health Research University of Southern Denmark Odense Denmark; ^3^ Research Support Unit Vejle Hospital, University Hospital of Southern Vejle Denmark; ^4^ Nuclear Medicine Department Vejle Hospital, University Hospital of Southern Vejle Denmark; ^5^ Department of Surgery Vejle Hospital, University Hospital of Southern Vejle Denmark

**Keywords:** cancer, human papillomavirus (HPV), population‐based cohort study, solid organ transplant recipient

## Abstract

Human papillomavirus (HPV) is a major cause of several epithelial cancers. Solid organ transplant recipients (SOTRs) are chronically immunosuppressed, but population‐based estimates of HPV‐related cancer risk across transplanted organs and by sex remain limited.

We conducted a nationwide, population‐based cohort study including all SOTRs in Denmark from January 1, 2000, to December 31, 2023. Information on transplantation and cancer diagnoses was obtained from the Danish National Patient Registry. Each SOTR was matched with five population controls by age, sex, and birth cohort. Incidence rates and hazard ratios (HRs) for HPV‐related cancers were estimated using Cox proportional hazards models, and cumulative incidence was calculated using the Aalen–Johansen method.

The cohort comprised 6509 SOTRs and 32,545 matched controls, followed for a median of 7.8 years (IQR 3.8–13.1). The 10‐year incidence rate of HPV‐related cancers was higher among SOTRs than controls (0.10 [95% CI 0.08–0.14] vs. 0.04 [95% CI 0.03–0.05] per 100 person‐years). The overall HR for any HPV‐related cancer was 2.49 (95% CI 1.74–3.56) and was highest for anogenital cancers (HR 3.29; 95% CI 1.88–5.76). Relative risks were higher among women than men (HR 3.11 [95% CI 1.77–5.46] vs. 2.15 [95% CI 1.35–3.43]), with cumulative incidence indicating earlier onset among women.

Solid organ transplant recipients experience a sustained two‐to‐three‐fold increased risk of HPV‐related cancers, with pronounced sex‐specific differences. These findings provide robust population‐level evidence of elevated long‐term cancer risk in immunosuppressed individuals.

## Abstract (highlights)

What's new?

Solid organ transplant recipients (SOTRs) face chronic immunosuppression, leaving them susceptible to viral infections. Of particular concern is persistent infection with human papillomavirus (HPV), which presents a significant cancer risk. This nationwide Danish cohort study estimated cancer risk in SOTRs using health registry data. Compared to matched controls, SOTRs exhibited a two‐ to three‐fold higher incidence of HPV‐related cancers, particularly anogenital cancers. Risk of anogenital cancer was highest among women, who also experienced earlier disease onset. The findings cast light on elevated long‐term cancer risks in transplant recipients, highlighting the relevance of targeted screening and monitoring strategies in immunosuppressed populations.

## Graphical abstract

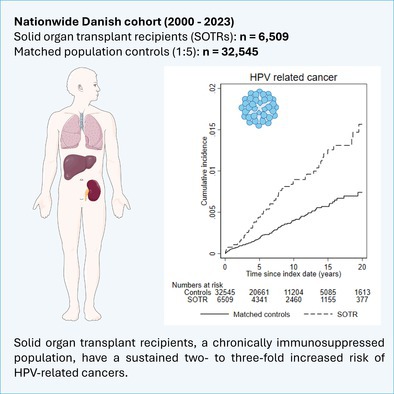

**Abbreviations**
95% CI: 95% confidence interval
HPV: Human papillomavirus
HR: Hazard ratio
ICD‐10: International Classification of Diseases, 10th Revision
IQR: Interquartile range
IR: Incidence rate
PYR: Person‐years
SIR: Standardized incidence ratio
SOTR: Solid organ transplant recipient

## 1 Introduction

Human papillomavirus (HPV) remains the most widespread sexually transmitted infection globally. Although the majority of infections are transient and resolve spontaneously within one to two years, persistent HPV infection can progress to precancerous lesions and ultimately lead to invasive malignancies. Alarmingly, the global burden of HPV‐associated cancers continues to rise despite preventive efforts [1].

Individuals with impaired immune function are particularly susceptible to persistent viral infections and virus‐associated malignancies. Among these, solid organ transplant recipients (SOTRs) represent a high‐risk population due to lifelong immunosuppressive therapy. Compared with the general population, SOTRs exhibit a markedly increased overall cancer incidence—approximately four‐fold—and a three‐ to five‐fold elevation in cancer‐related mortality [2]. This elevated risk is driven in part by the enhanced persistence and potential reactivation of latent oncogenic viruses, including HPV, under immunosuppressive conditions [3].

The oncogenic potential of HPV manifests across multiple anatomical sites, with SOTRs facing particularly high risks for anal squamous cell carcinoma. Reported standardised incidence ratios (SIRs) vary widely, ranging from 3 to over 100 [4]. Swedish registry data similarly document consistently elevated risks across organ types and cancer subtypes, underscoring the systemic vulnerability introduced by long‐term immunosuppression [5, 6].

For women, gynecological cancers such as cervical, vulvar, and vaginal cancers often occur at younger ages and within a few years of transplantation [7]. As survival rates post‐transplantation continue to improve, malignancy has emerged as a leading cause of late mortality among SOTRs—raising urgent questions about prevention and early detection.

Denmark provides a unique setting in which to address these questions. The country's extensive national health registries allow for high‐quality epidemiological tracking, while long‐standing preventive programs—such as universal cervical screening since the 1990s and the phased introduction of HPV vaccination (for girls in 2009 and boys in 2019)—offer an opportunity to assess real‐world impacts [8]. However, the effectiveness of these preventive measures in immunosuppressed populations remains poorly understood.

We aimed to quantify the risk of HPV‐related cancers among SOTRs in Denmark and to explore potential sex‐specific differences in cancer incidence compared with the general population. We focused on incident cases of oropharyngeal and anogenital cancer, comprising cervical, vaginal, vulvar, penile, and anal cancer.

## 2 Methods

### 2.1 Study Design and Data Sources

We conducted a nationwide, population‐based cohort study linking multiple Danish health registries through unique personal identifiers assigned to all residents since 1968 [9]. The Civil Registration System is continuously updated with information on vital status, migration, and residence.

Data on all hospital admissions, transplantations, and cancer outcomes were obtained from the Danish National Patient Registry [10]. Registry completeness is ensured through mandatory physician reporting and linkage with hospital discharge and mortality databases.

### 2.2 Study Population and Follow‐Up

**FIGURE 1:**
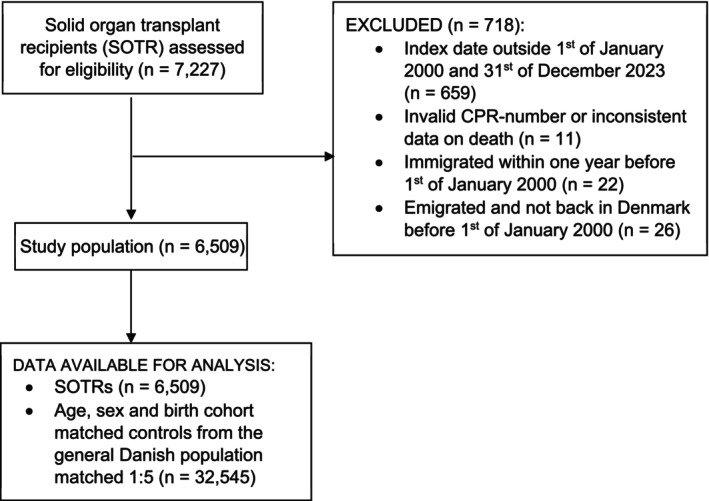
Flow chart of the study population.

The SOTR population was defined as all individuals undergoing kidney, pancreas, liver, heart, or lung transplantation between Jan 1, 2000 and Dec 31, 2023 (Figure 1). Each SOTR was matched to five population controls by age, sex, and birth cohort at the time of transplantation. Patients and controls with a prior history of HPV‐related cancer (oropharyngeal, cervical, vaginal, vulvar, penile, or anal) or with less than 1 year of residence were excluded. Follow‐up ended at the first occurrence of the outcome of interest, death, emigration, or Dec 31, 2023. Controls were censored at the end of follow‐up for their corresponding SOTR case to avoid including excess person‐time at risk.

### 2.3 Outcomes

The primary outcome was the first diagnosis of any HPV‐related cancer (oropharyngeal and anogenital cancer, comprising cervical, vaginal, vulvar, penile, and anal cancer). Cancer by location, specific cancers, and death were reported as secondary outcomes. All outcomes were identified by ICD‐10 codes listed in (Table S1).

### 2.4 Statistical Analysis

Descriptive statistics were expressed as counts (%) or medians (IQRs). Incidence rates (IRs) were calculated per 100 person‐years (pyr). Cumulative incidence functions were estimated via the Aalen–Johansen method considering death as a competing event. Hazard ratios (HRs) with 95% confidence intervals (CIs) were estimated using Cox proportional‐hazards regression stratified by match group.

The analyses were reported at 10 years of follow‐up overall and further stratified by sex, cancer location and organ type. All analyses were conducted in *STATA* v19.5 MP (StataCorp, College Station, TX, USA) with a two‐sided significance level of *p* < 0.05.

## 3 Results

This nationwide Danish cohort study comprised 6509 SOTRs, predominantly males with a median age of 50 years (IQR 38–59) at transplantation (Table 1). Kidney transplantation was most common (66.9%), followed by liver, lung and heart transplants. The matching by age, sex, and birth cohort, ensured comparable baseline characteristics between cases and controls. The proportion of SOTRs with prior (non‐HPV related) cancer was slightly higher than among the matched controls (6.7% vs. 3.7%). The median follow‐up among SOTRs was 7.8 years (IQR 3.8–13.1).

**TABLE 1:** Baseline characteristics of the study population: 6509 solid organ transplant recipients (SOTR) and sex, age and birth cohort matched controls (ratio 1:5).

	Solid organ transplant recipients (SOTR)	Matched controls	Total
N	6.509	32.545	39.054
Age, (median, IQR)	50 (38–59)	50 (38–59)	50 (38–59)
Sex, N (%female)	2.518 (38.7%)	12.510 (38.7%)	15.108 (38.7%)
Transplanted organ^a^, *N* (%)			
Heart	576 (8.8%)	—	576 (8.8%)
Lung	664 (10.1%)	—	664 (10.1%)
Liver	896 (13.6%)	—	896 (13.6%)
Kidney	4.397 (66.9%)	—	4.397 (66.9%)
Pancreas	41 (0.6%)	—	41 (0.6%)
Prior cancer, *N* (%yes)	438 (6.7%)	1.202 (3.7%)	1.640 (4.2%)
Years of follow‐up, (median, IQR)	7.8 (3.8–13.1)	7.2 (3.5–12.5)	7.3 (3.5–12.6)

^a^ In 65 patients, two organs were transplanted during the same surgical procedure and consequently, the total number of transplanted organs amounts to *N* = 6574.

Within 10 years of follow up, 46 HPV‐related cancers occurred among SOTRs compared with 86 among controls, corresponding to incidence rates of 0.10 [95% CI 0.08–0.14] and 0.04 [95% CI 0.03–0.05] per 100 person‐years, respectively (Table 2). The SOTR cancer cases had a median age of 58.8 (IQR 51.5–65.7) at diagnosis. Overall, SOTRs experienced a 2.5‐fold higher risk of HPV‐related malignancies than the background population (HR 2.49, [95% CI 1.74–3.56]; *p* < 0.001).

**TABLE 2:** Ten‐year incidence of HPV‐related cancers among solid organ transplant recipients (SOTR) reported as incidence rate (IR) per 100 person‐years and ten‐year cumulative incidence.

	Events (n)	Incidence rate (IR) per 100 pyr	Cumulative incidence at 10 years	Hazard ratio (HR), 95% CI	p
All HPV‐related cancers					
SOTR	46	0.10 (0.08–0.14)	0.009	2.49 (1.74–3.56)	< 0.001
Matched controls	86	0.04 (0.03–0.05)	0.004		
Cancer location					
*Oropharyngeal cancer*					
SOTR	26	0.06 (0.04–0.09)	0.005	2.14 (1.34–3.42)	< 0.001
Matched controls	56	0.03 (0.02–0.03)	0.003		
*Anogenital cancer*					
SOTR	21	0.05 (0.03–0.07)	0.001	3.29 (1.88–5.76)	< 0.001
Matched controls	30	0.01 (0.01–0.02)	0.004		
Specific cancer type					
*Cervical cancer*					
SOTR	5	0.03 (0.01–0.07)	0.002	1.54 (0.56–4.27)	0.404
Matched controls	15	0.02 (0.01–0.03)	0.002		
*Vaginal cancer*					
SOTR	< 5	0.01 (0.00–0.04)	0.001		
Matched controls	< 5				
*Vulvar cancer*					
SOTR	6	0.03 (0.02–0.08)	0.003	29.12 (3.50–242.05)	0.002
Matched controls	< 5	0.00 (0.00–0.01)	0.000		
*Penile cancer*					
SOTR	5	0.02 (0.01–0.04)	0.001	4.52 (1.30–15.71)	0.018
Matched controls	5	0.00 (0.00–0.01)	0.000		
*Anal cancer*					
SOTR	5	0.01 (0.00–0.03)	0.001	2.69 (0.90–8.03)	0.076
Matched controls	9	0.00 (0.00–0.01)	0.000		
Death					
SOTR	1565	3.54 (3.37–3.72)	0.296	5.27 (4.91–5.67)	< 0.001
Matched controls	1404	0.66 (0.63–0.70)	0.066		

*Note:* Relative occurrence compared with matched controls (1:5) is reported as hazard ratio (HR) with corresponding 95% CI.

**TABLE 2a:** Ten‐year incidence of HPV‐related cancers among male solid organ transplant recipients (SOTR) reported as incidence rate (IR) per 100 person‐years and ten‐year cumulative incidence.

Men (*N* = 23,946)	Events (n)	Incidence rate (IR) per 100 pyr	Cumulative incidence at 10 years	Hazard ratio (HR), 95% CI	*p*
All HPV‐related cancers					
SOTR	26	0.10 (0.07–0.14)	0.008	2.15 (1.35–3.43)	0.001
Matched controls	55	0.04 (0.03–0.06)	0.004		
Cancer location					
*Oropharyngeal cancer*					
SOTR	20	0.07 (0.05–0.12)	0.007	2.02 (1.19–3.42)	0.009
Matched controls	45	0.03 (0.03–0.05)	0.004		
*Anogenital cancer*					
SOTR	7	0.03 (0.01–0.05)	0.002	3.20 (1.22–8.45)	0.019
Matched controls	10	0.01 (0.00–0.01)	0.001		
Death					
SOTR	1000	3.70 (3.48–3.94)	0.308	4.80 (4.40–5.25)	< 0.001
Matched controls	978	0.76 (0.71–0.81)	0.075		

*Note:* Relative occurrence compared with matched controls (1:5) is reported as hazard ratio (HR) with corresponding 95% CI.

**FIGURE 2:**
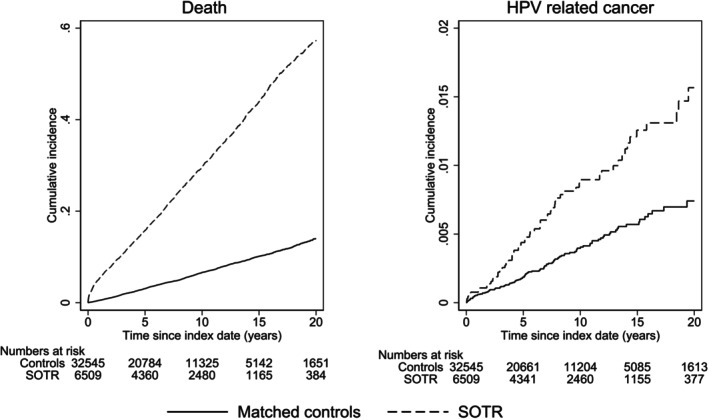
Cumulative incidence of mortality and HPV‐related cancers among solid organ transplant recipients and matched population controls, Denmark, 2000–2023. Cumulative incidence curves were estimated using the Aalen–Johansen method, with death and HPV‐related cancers as separate endpoints. Numbers at risk are shown below each plot. Solid organ transplant recipients (SOTRs) exhibited substantially higher cumulative incidence for both mortality and HPV‐related malignancies compared with matched population controls throughout follow‐up.

At ten years, the cumulative incidence of any HPV‐related malignancy reached 0.9% among SOTRs compared with 0.4% among controls (Table 2 and Figure 2). The cumulative incidence curves showed an early divergence within the first five years following transplantation and a persistent gap thereafter.

**FIGURE 3:**
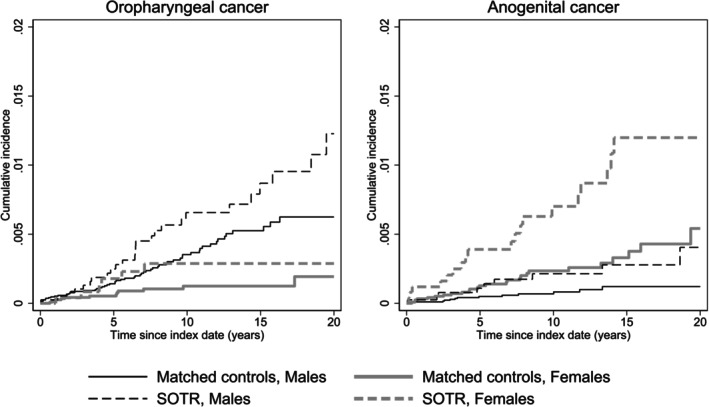
Cumulative incidence of oropharyngeal and anogenital cancers by sex among solid organ transplant recipients and matched population controls, Denmark, 2000–2023 Cumulative incidence of HPV‐related oropharyngeal and anogenital cancers was estimated using the Aalen–Johansen method, stratified by sex. Female transplant recipients showed earlier onset and steeper risk accumulation for anogenital cancers than males, whereas males had higher risk of oropharyngeal cancer.

When examined by anatomical site, both oropharyngeal and anogenital regions showed increased risks. The HR for oropharyngeal cancers was 2.14 [95% CI 1.34–3.42], whereas the HR for anogenital cancers was 3.29 [95% CI 1.88–5.76] (Table 2). Sex‐stratified analyses demonstrated clinically relevant differences, since women had a higher overall risk of HPV‐related cancers than men (female HR 3.11 [95% CI 1.77–5.46] vs. male HR 2.15 [95% CI 1.35–3.43], Tables 2, 2a, 2b), although the difference did not reach statistical significance (*p* = 0.34). Within cancer site, anogenital cancers tended to display a higher risk compared to control than oropharyngeal cancer, although anogenital cancer was more prevalent among females, whereas males had a higher risk of oropharyngeal cancer (Tables 2, 2a, 2b, Figure 3).

**TABLE 2b:** Ten‐year incidence of HPV‐related cancers among female solid organ transplant recipients (SOTR) reported as incidence rate (IR) per 100 person‐years and ten‐year cumulative incidence.

Women (*N* = 15,108)	Events (n)	Incidence rate (IR) per 100 pyr	Cumulative incidence at 10 years	Hazard ratio (HR), 95% CI	*p*
All HPV‐related cancers					
SOTR	20	0.12 (0.08–0.18)	0.010	3.11 (1.77–5.46)	< 0.001
Matched controls	31	0.04 (0.03–0.05)	0.003		
Cancer location					
*Oropharyngeal cancer*					
SOTR	6	0.03 (0.02–0.08)	0.002	2.69 (0.99–7.28)	0.051
Matched controls	11	0.01 (0.01–0.02)	0.001		
*Anogenital cancer*					
SOTR	14	0.08 (0.05–0.14)	0.007	3.34 (1.68–6.62)	< 0.001
Matched controls	20	0.02 (0.02–0.04)	0.002		
Death					
SOTR	565	3.28 (3.02–3.56)	0.276	6.36 (5.61–7.22)	< 0.001
Matched controls	426	0.51 (0.46–0.56)	0.051		

*Note:* Relative occurrence compared with matched controls (1:5) is reported as hazard ratio (HR) with corresponding 95% CI.

In addition, the ten‐year cumulative incidence of HPV‐related cancers varied markedly by transplanted organ, ranging from 0.007 in kidney recipients to 0.015 in liver and 0.013 in lung recipients. Compared with matched controls, the risk was highest among lung (HR 11.18 [95% CI 2.89–43.26]) and liver transplant recipients (HR 4.51 [1.87–10.90]), whereas kidney recipients had a more modest but still significant elevation (HR 1.97 [1.24–3.11]) (Table S2).

## 4 Discussion

In this nationwide cohort study, solid organ transplant recipients had a substantially elevated risk of HPV‐related cancers. Importantly, we observed a pronounced sex difference in the risk of HPV‐related malignancies, with women exhibiting substantially higher cumulative incidence and hazard ratios than men across all cancer sites. These findings underscore the apparent increased susceptibility of female transplant recipients to HPV‐driven carcinogenesis, even in a setting with established national cervical cancer screening.

The excess risk among women was particularly evident for anogenital cancers, including cervical, vulvar, and vaginal malignancies, consistent with prior U.S. and Swedish studies [3, 7]. However, the magnitude of difference observed in our cohort appears greater than previously reported, suggesting potential cumulative effects of longer post‐transplant survival and chronic immunosuppression.

Male recipients also faced an elevated risk, particularly for anal and oropharyngeal squamous cell carcinomas, though absolute incidence remained lower than in women. This pattern likely reflects both differences in viral exposure pathways and the influence of pre‐existing screening programs, which in Denmark have historically targeted women. The absence of structured HPV screening or vaccination catch‐up for adult males may further contribute to residual risk.

The variation in HPV‐related cancer risk observed across transplanted organs may plausibly be related to differences in immunosuppressive treatment intensity. In Denmark, lung and liver transplant recipients are generally managed with more intensive and sustained immunosuppressive regimens than kidney recipients, often involving higher cumulative exposure and more frequent use of induction therapy. Such treatment patterns may promote viral persistence and reactivation and impair immune surveillance, thereby increasing susceptibility to HPV‐driven carcinogenesis. Although information on dispensed immunosuppressive medications is partly available through national prescription registries, immunosuppressive therapy administered in hospital settings may be incompletely captured. A comprehensive evaluation of treatment‐specific effects would therefore require a dedicated study with detailed longitudinal data on immunosuppressive regimens, which was beyond the scope of the present population‐based analysis. Our study extends prior registry‐based evidence by providing sex‐stratified risk estimates across multiple organ types within a universal healthcare system. By leveraging linkage between the Danish National Patient Registry and Cancer Registry, we achieved near‐complete follow‐up, minimizing bias from loss to follow‐up. Compared with previous work limited to kidney recipients or shorter follow‐up, our data capture contemporary transplantation cohorts exposed to modern immunosuppressive regimens [5].

Although the sex‐stratified analyses did not reach statistical significance, women had a higher overall risk of HPV‐related cancers than men, suggesting that biological differences between sexes may still influence HPV‐associated cancer risk among transplant recipients. Female SOTRs are more likely to have had prior anogenital HPV infection, which may serve as a reservoir for persistent infection. Interpretation of these sex‐specific patterns should also consider that the attributable fraction of HPV differs across anogenital cancer sites, ranging from nearly 100% for cervical cancer to substantially lower levels for some male‐specific cancers. Despite Denmark's comprehensive HPV prevention infrastructure, most notably childhood vaccination and population‐based cervical screening, the persistently elevated cancer risk observed in SOTRs suggests that such general strategies may not adequately protect immunosuppressed individuals. However, caution is warranted when interpreting the potential impact of HPV vaccination, as the full effect is unlikely to be reflected in these findings, early vaccine uptake was relatively low, and although some vaccinated women and men may be included, most have not yet reached the age at which they would be represented in this cohort. The findings emphasize the need for adaptive post‐transplant surveillance strategies, potentially including HPV testing of both sexes before and after transplantation and the evaluation of vaccine booster efficacy among long‐term SOTRs.

Strengths of this study include its nationwide design, high data completeness, and sex‐stratified analyses. This study has several limitations. First, we lacked access to detailed data on individual immunosuppressive treatment regimens and HPV‐vaccination, which may have influenced the risk of HPV‐related carcinogenesis. Second, information on HPV genotypes was not available, limiting the ability to assess vaccine coverage or the potential need for booster strategies. Third, data on behavioral risk factors—such as smoking, sexual practices, and participation in screening programs—were unavailable, potentially confounding the observed associations between transplant recipients and the general population. Fourth, although a history of prior cancer was more common among SOTRs, the risk of subsequent HPV‐related cancer was similar in those with and without prior cancer (1.8% vs. 1.7%, *p* = 0.8), although the small number of events limits the precision of this comparison.

## 5 Conclusion

This population‐based study identifies a persistent excess risk of HPV‐related cancers in solid organ transplant recipients, extending across transplanted organs and differing by sex and cancer site. The observed risk patterns suggest that HPV‐associated malignancies represent a clinically relevant aspect of survivorship after transplantation. These findings provide a basis for future research to inform clinical approaches to cancer prevention and follow‐up in immunosuppressed individuals.

## Author Contributions

**Signe Timm:** methodology, investigation, formal analysis, visualization, writing – original draft, writing – review and editing. **Flemming Skjøth:** methodology, data curation, investigation, formal analysis, visualization, writing – original draft, writing – review and editing. **Torben Frøstrup Hansen:** investigation, funding acquisition, writing – review and editing. **Lars Henrik Jensen:** investigation, funding acquisition, writing – review and editing. **Paw Christian Holdgaard:** conceptualization, investigation, writing – review and editing. **Lars Ulrik Fokdal:** conceptualization, investigation, writing – review and editing. **Torben Bjerregaard Larsen:** conceptualization, investigation, writing – original draft, writing – review and editing. **Mette Moeller Soerensen:** conceptualization, investigation, writing – original draft, writing – review and editing.

## Funding

Supported by internal institutional funding, the funding source had no role in study design, data collection, analysis, interpretation, or writing.

## Conflicts of Interest

The authors declare no conflicts of interest.

## Supporting information


**Table S1:** Data sources.
**Table S2:** Ten‐year incidence of HPV‐related cancers among solid organ transplant recipients (SOTR) stratified by.

## Data Availability

Data access was provided through the Open Patient data Explorative Network (OPEN), Odense University Hospital, Region of Southern Denmark, and Statistics Denmark. The study was approved by the Danish Data Protection Agency via institutional registration with the Region of Southern Denmark (reference 23‐16036). Access to Danish health data requires affiliation or formal collaboration with a Danish research institution; prior authorization from relevant data holders; and secure research environments without data transfer or export for external use. Other data that support the findings of this study are available from the corresponding author upon reasonable request.
