# Supplementary material for: HPV‐Related Cancers in Solid Organ Transplant Recipients: A Nationwide Danish Cohort Study
Source: Int J Cancer. 2026 Apr 28;159(6):1432–9. doi: 10.1002/ijc.70529 (PMC13397293; doi:10.1002/ijc.70529)
Supplement: Supplementary file 1 — Table S1: Data sources. Table S2: Ten‐year incidence of HPV‐related cancers among solid organ transplant recipients (SOTR) stratified by. [file IJC-159-1432-s001.pdf]

HPV-related Cancers in Solid Organ Transplant Recipients: A Nationwide Danish Cohort Study

Signe Timm, Flemming Skjøth, Torben Frøstrup Hansen, Lars Henrik Jensen, Paw Christian Holdgaard, Lars Ulrik Fokdal, Torben Bjerregaard Larsen, Mette Moeller Soerensen.

Supplementary material: Tables S1 and S2

Table S1: Data sources

Data source	Data		Code
Danish National Patient Registry	Diagnosis (IDC-10)	Anale cancer	C21
		Cervical cancer	C53
		Vulvar cancer	C51
		Penile cancer	C60 C680
		Vaginal cancer	C52
		Oral cavity cancer	C00 C01 C02 C03 C04 C05 C06 C07 C08
		Farynx cancer	C09 C10
		Larynx cancer	C32
		Cancer (excluding C44)	C0 C1 C2 C3 C40 C41 C42 C43 C45 C46 C47 C48 C49 C5 C6 C7 C8 C9
	Operation (SKS)	Heart transplant	KFQA
		Lung transplant	KGDG
		Heart and lung transplant	KFQB
		Liver transplant	KJJC
		Kidney transplant	KKAS
		Pancreas transplant	KJLE

Table S2: Ten-year incidence of HPV-related cancers among solid organ transplant recipients (SOTR) stratified by transplanted organ reported as incidence rate (IR) per 100 person-years and ten-year cumulative incidence. Relative occurrence compared with matched controls (1:5) is reported as hazard ratio (HR) with correponding 95% CI.

	**Events (n)**	**Incidence rate (IR) per 100 pyr**	**Cumulative Incidence at 10 years**	**Hazard ratio (HR), 95% CI**	**p**
All HPV-related cancers					
Heart transplant					
SOTR	< 5	0.08 (0.03-0.24)	0.008	1.25 (0.35-4.48)	0.737
Matched controls	11	0.06 (0.03-0.11)	0.005		
Kidney transplant					
SOTR	26	0.08 (0.06-0.12)	0.007	1.97 (1.24-3.11)	0.004
Matched controls	62	0.04 (0.03-0.05)	0.004		
Liver transplant					
SOTR	10	0.18 (0.10-0.34)	0.015	4.51 (1.87-10.90)	< 0.001
Matched controls	10	0.04 (0.02-0.07)	0.003		
Lung transplant					
SOTR	7	0.20 (0.10-0.43)	0.013	11.18 (2.89-43.26)	< 0.001
Matched controls	< 5	0.02 (0.01-0.06)	0.001		
